# Complex Patterns of Cannabinoid Alkyl Side-Chain Inheritance in *Cannabis*

**DOI:** 10.1038/s41598-019-47812-2

**Published:** 2019-08-06

**Authors:** Matthew T. Welling, Lei Liu, Carolyn A. Raymond, Tobias Kretzschmar, Omid Ansari, Graham J. King

**Affiliations:** 1 Southern Cross Plant Science, Southern Cross University, Lismore, New South Wales 2480 Australia; 2 Ecofibre Ltd, Brisbane, Queensland 4014 Australia; 3 Ananda Hemp Ltd, Cynthiana, Kentucky 41031 USA

**Keywords:** Biologics, Secondary metabolism, Natural variation in plants

## Abstract

The cannabinoid alkyl side-chain represents an important pharmacophore, where genetic targeting of alkyl homologs has the potential to provide enhanced forms of *Cannabis* for biopharmaceutical manufacture. Delta(9)-tetrahydrocannabinolic acid (THCA) and cannabidiolic acid (CBDA) synthase genes govern dicyclic (CBDA) and tricyclic (THCA) cannabinoid composition. However, the inheritance of alkyl side-chain length has not been resolved, and few studies have investigated the contributions and interactions between cannabinoid synthesis pathway loci. To examine the inheritance of chemical phenotype (chemotype), *THCAS* and *CBDAS* genotypes were scored and alkyl cannabinoid segregation analysed in 210 F_2_ progeny derived from a cross between two *Cannabis* chemotypes divergent for alkyl and cyclic cannabinoids. Inheritance patterns of F_2_ progeny were non-Gaussian and deviated from Mendelian expectations. However, discrete alkyl cannabinoid segregation patterns consistent with digenic as well as epistatic modes of inheritance were observed among F_2_ *THCAS* and *CBDAS* genotypes. These results suggest linkage between cannabinoid pathway loci and highlight the need for further detailed characterisation of cannabinoid inheritance to facilitate metabolic engineering of chemically elite germplasm.

## Introduction

*Cannabis* is a phylogeographically divergent^1^ notably heterozygote^2^ anemophilous (wind pollinated) angiosperm genus^3^, which has undergone sub-selection for fibre, seed^4^, recreational drug, and medical end-uses^5,6^. Despite a long history of domestication dating back several thousand years^7^, exploitation of *Cannabis ex situ* genetic resources using modern improvement strategies has been hampered due to legal constraints relating to the plant’s status as a narcotic^8^.

*Cannabis* plants produce a class of therapeutically important isoprenylated resorcinyl polyketides^9^, more commonly identified as (phyto)cannabinoids^10^. These accumulate predominantly within capitate stalked trichromes on floral tissues^11^. Cannabinoids are synthesised with a carboxylated resorcinyl core, which readily decarboxylates by non-enzymatic means^12^. Structurally, cannabinoids vary by isoprenyl topological arrangement^13^, of which dicyclic cannabidiol (CBD)-type and tricyclic delta(9)-tetrahydrocannabinol (THC)-type cannabinoids are commonly encountered *in planta*^14^. Another important structural feature of cannabinoids is the resorcinyl alkyl side-chain which typically occurs in either pentyl (C_5_) or to a lesser extent propyl (C_3_) configuration^15,16^, although a variety of odd and even carbon lengths have been reported as minor constituents in a subset of germplasm^17,18^.

The G-protein-coupled cannabinoid type 1 (CB_1_R) and 2 (CB_2_R) receptors are principally implicated in mediating biological effects of the human endocannabinoid system, a complex aggregate of several therapeutic targets, multiple signalling pathways and ion channels^19,20^. The pro-homeostatic functionality of the endocannabinoid system is thought to stem from its secretory regulation of signalling molecules^20^, namely various neurotransmitters (e.g. 5-HT and GABA)^21,22^ and cytokines (e.g. TNF-α and IL-17)^23,24^. Associated neuro-immunomodulatory activity by exogenous cannabinoid ligands appear beneficial in a myriad of seemingly unrelated indications, ranging from the treatment of seizures in refractory paediatric epilepsies (Epidiolex^®^)^25^ through to chronic pain in advanced cancer patients (Nabiximols)^26^. Structure-activity relationship studies have identified the resorcinyl alkyl group as a critical pharmacophoric element^27,28^. Elongation of the carbon side-chain increases cannabinoid receptor binding affinity^29,30^, with pharmacological potency of C_4_ to C_8_ alkyl chain homologs showing systematic increases up to 29-fold^30^. Despite the potential for metabolic engineering of the alkyl group for *in planta* therapeutic cannabinoid portfolio expansion^15,31^, uncertainty over the genetic and biosynthetic regulation of alkyl cannabinoid homology hinders the development of novel recombinant cannabinoid breeding lines for biopharmaceutical exploitation.

The cannabinoid structural motif is generated from substrates originating from two independent biosynthetic pathways. Aromatic prenylation of geranyl diphosphate (GPP) and a phenolic alkylresorcinolic acid intermediate form monocyclic cannabinoids that feature a linear isoprenyl residue (e.g. cannabigerolic acid (CBGA))^32,33^. Chain length of the alkylresorcinol fatty acid (FA) starter unit is thought to determine alkyl cannabinoid homology^34,35^. This hypothesis has been supported using a synthetic cell-free enzymatic platform which produced the propyl-cannabinoid intermediate cannabigerovarinic acid (CBGVA) from a C_3_ alkylresorcinol substrate (divarinic acid)^36^. *In vivo* production of CBGVA and divarinic acid as well as associated end products delta(9)-tetrahydrocannabivarinic acid (THCVA) and cannabidivarinic acid (CBDVA) have also recently been reported in engineered yeast strains fed the predicted C_3_ alkyl cannabinoid intermediate butanoyl-CoA^37^. However, resolution of associated *in planta* biosynthetic pathways has largely focused on C_5_ alkyl species^33,38^.

Cannabidiolic acid synthase (CBDAS) and delta(9)-tetrahydrocannabinolic acid synthase (THCAS) perform stereoselective oxidative cyclisation of the isoprenyl moiety, forming dicyclic and tricyclic cannabinoids. Physical and genetic mapping of *THCAS* and *CBDAS* genes has recently allowed for alignment of genetic loci to resolve the cluster of closely-linked genes. These genomic regions appeared abundant with retrotransposable elements as well as pseudogenic tandem repeats, and their positions have been assigned within a larger low recombining pericentromeric gene-poor region^39,40^. Regions also appeared non-homologous between chemotypes which suggests significant divergence between chemotypic lineages, although the reported hemizygosity for *THCAS* and *CBDAS* may be an artefact of genome assembly due to the underlying complexity of this region^39,40^. While the presence of tandem *THCAS* as well as *CBDAS* arrays would imply oligogenic inheritance, genepool representative germplasm segregate in a 1:2:1 dicyclic: tricyclic cannabinoid ratio characteristic of a single codominant locus *B* model^41,42^. This suggests cannabinoid synthase tandem arrays may include functionally superfluous repeats which seldom recombine, that although separated in terms of physical distance (>1 Mbp)^40^, segregate in a manner that resembles mutually exclusive *B*_THCAS_ (*THCAS*) and *B*_CBDAS_ (*CBDAS*) alleles.

The dioecious reproduction of *Cannabis* often confounds genetic analysis. Previous analysis of tricyclic chemotypes segregating for alkyl cannabinoid composition inferred a multiple locus *A*^1^-*A*^2^-… *A*^n^ model, whereby alleles *A*_pr_^1−n^ and *A*_pe_^1−n^ with additive effect govern the proportion of alkyl cannabinoid homologs^31^. However, chemotypic continuity of the available progeny precluded demarcation of categories, thereby preventing chi-square analysis to resolve the inheritance model. To examine alkyl cannabinoid loci and determine their allelic assortment with cannabinoid synthase genes, we analysed a population segregating for alkyl and cyclic cannabinoid composition. Biparental reciprocal crosses between chemotypes divergent for alkyl and cyclic cannabinoids were performed, generating F_1_ hybrid families. A single F_2_ generation derived from an F_1_ male and female cross was developed for chemotypic segregation analysis. Cannabinoid profiling of F_2_ progeny along with genotypic analysis using a *THCAS*- and *CBDAS*-specific DNA sequence characterised amplified region (SCAR) marker assay was conducted to investigate interactions between cannabinoid pathway loci. Frequency distributions were determined using kernel density estimation, a statistical method of applying smoothing to a frequency histogram^43^. Kernel density was used to estimate underlying distributions and to demarcate chemotypes objectively into categories, thereby exposing modes of inheritance for alkyl side-chain length.

## Results

### Parental selection

**Figure 1:**
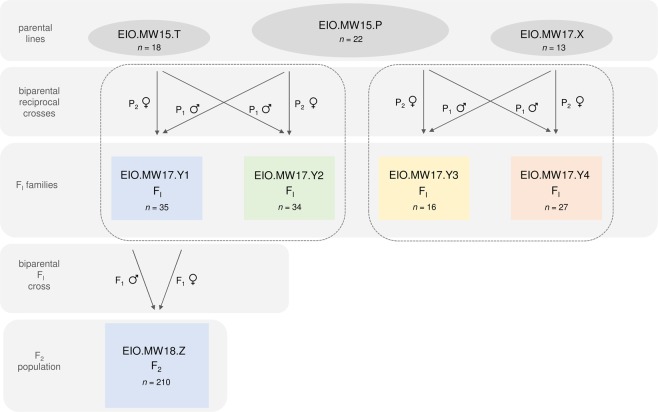
Schematic diagram of filial generations. Parental breeding lines were screened for cannabinoid composition and eight plants high in either F_C3_ as well as F_tricyclic_ or F_C5_ as well as F_dicyclic_ values served as parents for two biparental reciprocal crosses, generating four F_1_ hybrid families. A single male and female plant from the F_1_ hybrid family which demonstrated the highest level of F_C3_/F_C5_ homogeneity served as parents of an F_2_ population segregating for F_C3_/F_C5_ and F_dicyclic_/F_tricyclic_ cannabinoid composition. C_5_ alkyl cannabinoid fraction (F_C5_); C_3_ alkyl cannabinoid fraction (F_C3_); dicyclic cannabinoid fraction (F_dicyclic_); and tricyclic cannabinoid fraction (F_tricyclic_).

Juvenile plants of three parental lines were screened for cannabinoid composition. C_3_/C_5_ alkyl cannabinoid fractions (F_C3_/F_C5_) associated with alkyl cannabinoid loci (*A*^n^ loci) as well as di-/tri-cyclic cannabinoid fractions (F_dicyclic_/F_tricyclic_) associated with the *B* locus complex were determined from the fresh weight (*w/w*) cannabinoid content of CBDVA, THCVA, cannabidiolic acid (CBDA) and delta(9)-tetrahydrocannabinolic acid (THCA). Eight individual plants which exhibited either [high F_C3_ + F_tricyclic_ (e.g. THCVA)] or [high F_C5_ + F_dicyclic_ (e.g. CBDA)] cannabinoid chemotypes were tentatively assigned homozygote status at the *A* and *B* locus complexes (Table 1). These plants from accessions EIO.MW15.P (*n* = 4), EIO.MW15.T (*n* = 2) and EIO.MW17.X (*n* = 2) were selected as parents to generate two biparental reciprocal crosses, forming four F_1_ hybrid families (Fig. 1). Parents of F_1_ hybrid family EIO.MW17.Y1 exhibited the largest divergence in F_C3_ (Table 1). To further examine parental homozygosity in this lineage, P1 (EIO.MW15.P [07]) and P2 (EIO.MW15.T [02]) were scored using a codominant locus *B* DNA sequence characterised amplified region (SCAR) marker assay. As expected, P1 and P2 had a marker genotype homozygote for *THCAS* (*B*_THCAS_*B*_THCAS_) and homozygote *CBDAS* (*B*_CBDAS_*B*_CBDAS_), respectively.

**Table 1:** Experimental populations and chemotypic segregation between tricyclic C_3_ alkyl (THCVA) and dicyclic C_5_ alkyl (CBDA) *Cannabis* plants.

Cross	Generation	*n*	Sample ID	F_C3_ (% total)	F_dicyclic_ (% total)
EIO.MW15.P x EIO.MW15.T	P1	22	EIO.MW15.P [**07**]	69.8–92.9 [**88.3**]	0.1–2.0 [**0.9**]
	P2	18	EIO.MW15.T [**02**]	0.5–1.0 [**0.8**]	60.9–96.2 [**96.1**]
	F_I_	35	EIO.MW17.Y1 [**15**, **32**]	24.3–56.0 [**35.5**, **24.3**]	49.5–75.8 [**62.2**, **75.8**]
	F_2_	210	EIO.MW18.Z	0.7–88.0	0.1–95.9
EIO.MW15.T x EIO.MW15.P	P1	18	EIO.MW15.T [**04**]	0.5–1.0 [**0.8**]	60.9–96.2 [**95.0**]
	P2	22	EIO.MW15.P [**11**]	69.8–92.9 [**83.9**]	0.1–2.0 [**0.4**]
	F_I_	34	EIO.MW17.Y2	7.4–42.3	48.0–74.8
EIO.MW17.X x EIO.MW15.P	P1	13	EIO.MW17.X [**05**]	0.8–1.2 [**0.9**]	93.7–96.5 [**95.7**]
	P2	22	EIO.MW15.P [**18**]	69.8–92.9 [**86.8**]	0.1–2.0 [**1.3**]
	F_I_	16	EIO.MW17.Y3	25.0–64.8	56.2–66.2
EIO.MW15.P x EIO.MW17.X	P1	22	EIO.MW15.P [**03**]	69.8–92.9 [**87.2**]	0.1–2.0 [**0.3**]
	P2	13	EIO.MW17.X [**12**]	0.8–1.2 [**0.9**]	93.7–96.5 [**96.5**]
	F_I_	27	EIO.MW17.Y4	28.5–73.3	26.2–70.8

Accessions EIO.MW15.P, EIO.MW15.T and EIO.MW17.X sourced from the Ecofibre Ltd Global Germplasm Collection (EFGGC); Bold indicates parental sample ID and cannabinoid values. C3 alkyl cannabinoid fraction (F_C3_); dicyclic cannabinoid fraction (F_dicyclic_); delta(9)-tetrahydrocannabivarinic acid (THCVA); cannabidiolic acid (CBDA).

### F_1_ hybrid chemotypic uniformity

**Figure 2:**
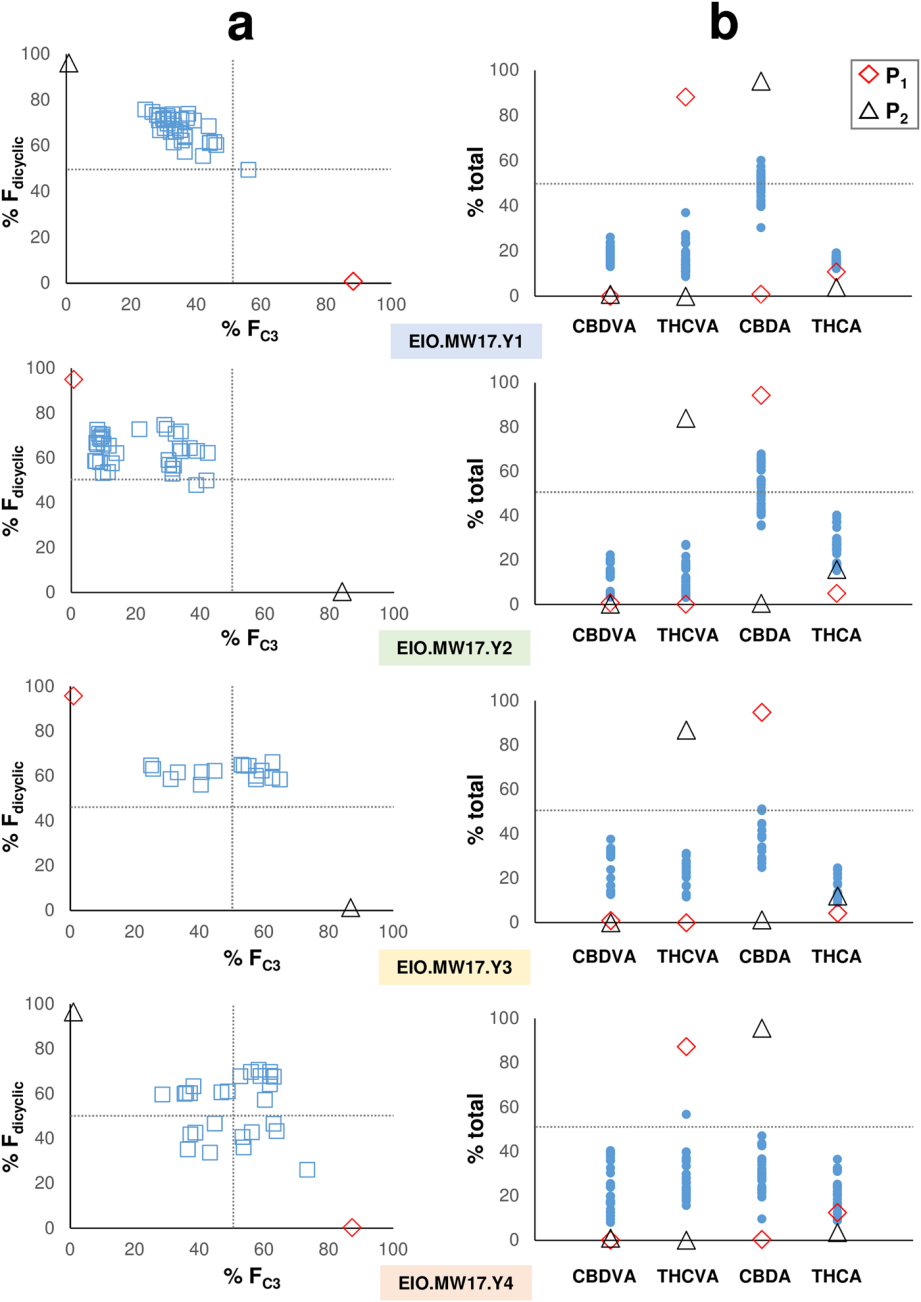
Chemotypic distributions of four F_1_ hybrid families. (**a**) Chemotypic distribution patterns of dicyclic and C_3_ alkyl cannabinoid composition within the total cannabinoid fraction. F_1_ chemotypes are intermediate to the parents, although discrete linage-specific distribution patterns are evident between families. (**b**) Compositional range of cannabinoids from individual plants within F_1_ hybrid families. *Blue squares* represent chemotypes of individual plants within F_1_ families; *Blue circles* represent cannabinoid composition of individual plants within F_1_ families; *Red diamond* represent female parent (P_1_); *Black triangle* represent male parent (P_2_); C_3_ alkyl cannabinoid fraction (F_C3_); dicyclic cannabinoid fraction (F_dicyclic_).

F_1_ individuals across all four hybrid families appeared chemotypically intermediate to the parents, although F_C3_/F_C5_ as well as F_dicyclic_/F_tricyclic_ distribution patterns were not uniform between families (Fig. 2a). No consistent maternal or paternal patterns of inheritance were observed for F_C3_ values among the reciprocal crosses. However, discrete lineage-specific chemotypic distribution patterns were evident, with F_1_ hybrid families (EIO.MW17.Y1, EIO.MW17.Y2) from EIO.MW15.T parents displaying cannabinoid composition skewed towards high F_C5_ as well as F_dicyclic_ values (CBDA) (Fig. 2a,b). Individuals within hybrid families displayed transgressive segregation for a subset of cannabinoids. CBDVA and THCA proportions (%/total) were greater than parent values, with CBDVA increasing by more than 20-fold (Fig. 2b). F_C3_/F_C5_ variance differed between the four F_1_ hybrid families (Table 2), with plants from EIO.MW17.Y1 having the least (Table 2). This, along with the *B* locus homozygote genotypes of EIO.MW17.Y1 parents, was interpreted as an indication of P1 and P2 homozygosity at the *A* locus complex. Single male and female plants of EIO.MW17.Y1 were crossed and alkyl cannabinoid segregation assessed in the resulting F_2_ generation (Fig. 1).

**Table 2:** Homogeneity of variances for four hybrid F_1_ families segregating for alkyl cannabinoid composition.

F_1_ family	Variance (F_C3_)	*df*
EIO.MW17.Y1	44.8^a^	34
EIO.MW17.Y2	160.4^a,b^	33
EIO.MW17.Y3	185.2^a,b^	15
EIO.MW17.Y4	138.0^a,b^	26

^a^Bartlett’s test for homogeneity of variances χ^2^ 15.34 on 3 *df* (*p* = 0.002); ^b^ Bartlett’s test for homogeneity of variances χ^2^ 0.42 on 2 *df* (*p* = 0.810); χ^2^ threshold for H_0_ acceptance at *df* = 3 and *df* = 2 (*p* = 0.05) is 7.815 and 5.991, respectively; C_3_ alkyl cannabinoid fraction (F_C3_).

### Inheritance patterns of the F_2_ progeny

**Figure 3:**
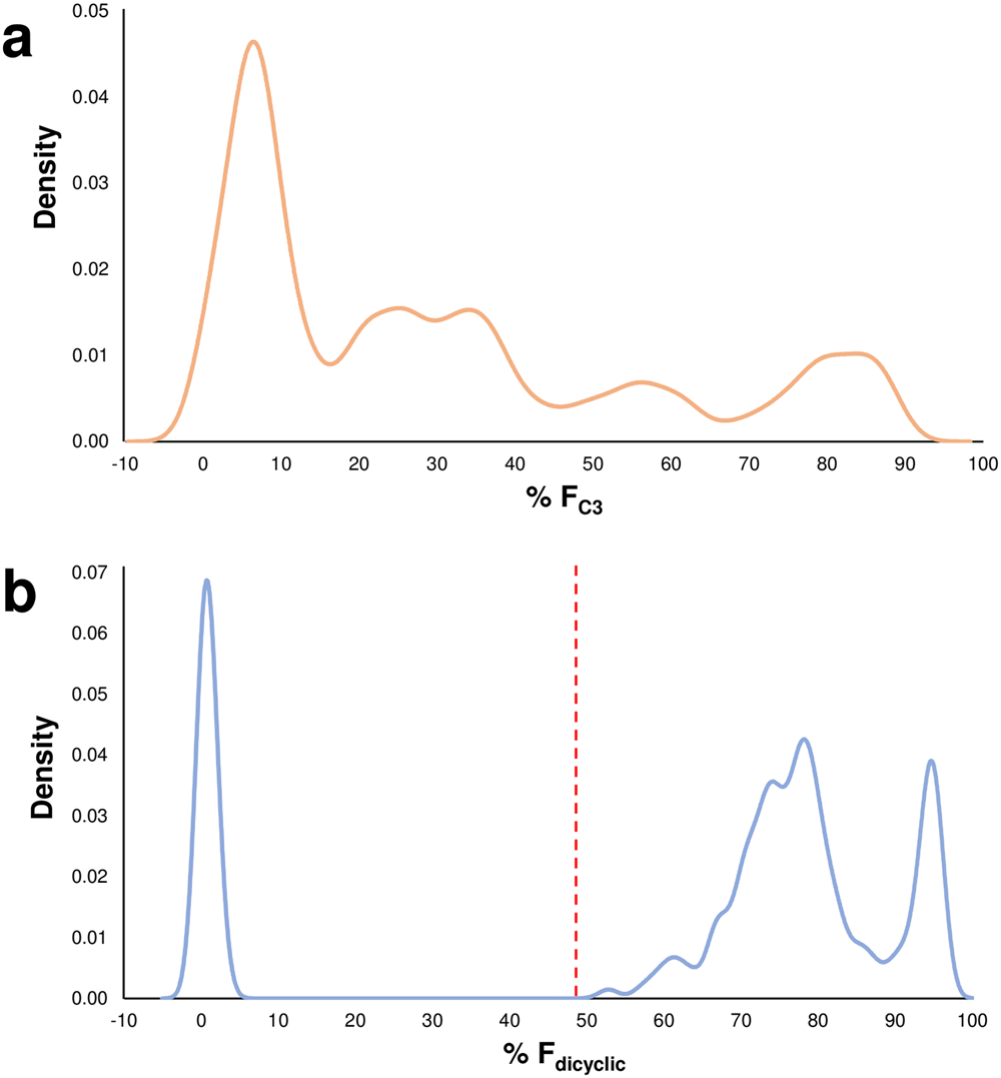
Chemotypic distribution patterns of F_2_ progeny segregating for cyclic and alkyl cannabinoid composition. (**a**) Kernel density estimates of F_C3_ values showing a pentapartite alkyl cannabinoid distribution. (**b**) Kernel density estimates of F_dicyclic_ values showing a predominantly tripartite cyclic cannabinoid distribution. Grid reference points for kernel density estimates for F_C3_ as well as F_dicyclic_ values are shown on the *x*-axes. For F_C3_ values, frequency distributions are skewed towards low F_C3_. The intermediate F_dicyclic_ distribution deviates from the mid-parent value of 48.5 and is skewed towards high F_dicyclic_. *Red line* indicates F_dicyclic_ mid-parent value; C_3_ alkyl cannabinoid fraction (F_C3_); dicyclic cannabinoid fraction (F_dicyclic_).

A continuous distribution of F_C3_/F_C5_ values was observed among the F_2_ progeny (Fig. 3a,b). To minimise classification error, kernel density estimates (KDE) were used to categorise individual plants objectively prior to testing the fit of genetic models. F_C3_/F_C5_ values for the F_2_ population were non-Gaussian and instead formed discrete pentapartite distributions. F_C3_/F_C5_ values were skewed towards low F_C3_ and deviated significantly from the expected 1:4:6:4:1 chemotypic segregation ratio (Fig. 3a, Table 3). KDE of F_dicyclic_/F_tricyclic_ values formed a predominantly tripartite distribution quasi-compatible with incomplete dominance and a 1:2:1 segregation ratio (Fig. 3b). However, discrete distributions embedded within the intermediate F_dicyclic_/F_tricyclic_ chemotypes suggested the possibility of additional F_dicyclic_/F_tricyclic_ categorises (Fig. 3b). The F_dicyclic_/F_tricyclic_ intermediate chemotypic distribution was skewed towards high F_dicyclic_ and diverged significantly from the mid-parent F_dicyclic_ value of 48.5 (Fig. 3b). The continuous distribution of F_dicyclic_ intermediate and high F_dicyclic_ values also prevented accurate dissection of inclusion/exclusion boundaries for chemotypic frequency estimation (Fig. 3b).

**Table 3:** Goodness-of-fit tests for alkyl cannabinoid chemotypic segregation ratios.

F_2_ population	F_C3_ categorisation (low – high)						Model		Goodness-of-fit				
Genotype	*n*	I	II	III	IV	V	No. loci	Gene effect	Expected ratio	χ^2^	*df*	Critical value^a^	H_0_ accepted
All	210	92	33	33	21	31	2	Additive	1:4:6:4:1	551.07	4	9.49	No
*B*_CBDAS_ *B*_CBDAS_	44	26	6	7	5	—	2	Dominant	9:3:3:1	2.71	3	7.82	Yes
*B*_THCAS_ *B*_CBDAS_	116	44	42	9	21	—	2	Dominant	9:3:3:1	59.33	3	7.82	No
*B*_THCAS_ *B*_THCAS_	50	21	22	7	—	—	2	Dominant, partially dominant	7:6:3	1.20	2	5.99	Yes

^a^Upper-tail critical values of the chi-square (χ^2^) distribution at *P* = 0.05; C_3_ alkyl cannabinoid fraction (F_C3_); locus *B* genotypes: homozygote *THCAS* (*B*_THCAS_*B*_THCAS_), homozygote *CBDAS* (*B*_CBDAS_*B*_CBDAS_), heterozygote *THCAS CBDAS* (*B*_THCAS_*B*_CBDAS_).

### Locus *B* genotype-specific alkyl cannabinoid distributions

**Figure 4:**
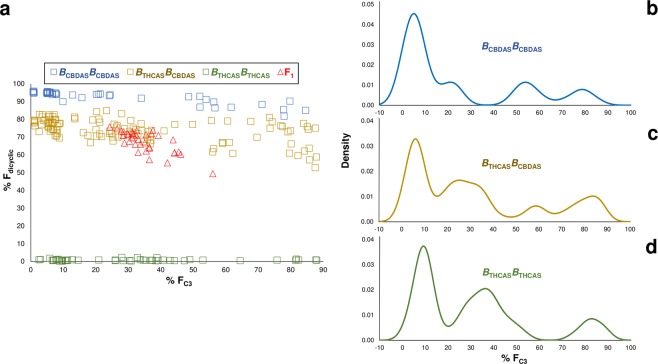
Locus *B* genotype-specific alkyl cannabinoid distribution patterns within F_2_ progeny. (**a**) Cyclic and alkyl cannabinoid inheritance patterns associated with locus *B* genotypes. (**b**) Kernel density estimates for homozygote *B*_CBDAS_*B*_CBDAS_ genotypes. (**c**) Kernel density estimates for heterozygote *B*_THCAS_*B*_CBDAS_ genotypes. (**d**) Kernel density estimates for homozygote *B*_THCAS_*B*_THCAS_ genotypes. Comparison of F_dicyclic_ values on the *y*-axes and F_C3_ values on the *x*-axes in Fig. 4a reveal three divergent F_C3_ inheritance patterns. Locus *B* genotypes are consistent with F_dicyclic_ values. C_3_-alkyl cannabinoid fraction (F_C3_); dicyclic cannabinoid fraction (F_dicyclic_); locus *B* genotypes: homozygote *THCAS* (*B*_THCAS_*B*_THCAS_), homozygote *CBDAS* (*B*_CBDAS_*B*_CBDAS_), heterozygote *THCAS CBDAS* (*B*_THCAS_*B*_CBDAS_).

To resolve F_dicyclic_/F_tricyclic_ chemotypic categories, the F_2_ progeny and F_1_ parents were genotyped for F_dicyclic_ (*CBDAS*) F_tricyclic_ (*THCAS*) associated alleles using the locus *B* DNA SCAR marker assay. The F_1_ parents had the predicted heterozygote *THCAS CBDAS* (*B*_THCAS_*B*_CBDAS_) genotypes. Genotypes *B*_CBDAS_*B*_CBDAS_, *B*_THCAS_*B*_CBDAS_ and *B*_THCAS_*B*_THCAS_, were consistent with the F_dicyclic_/F_tricyclic_ chemotype distributions in the F_2_ progeny (Fig. 4a). On the basis of genotypic frequency, a segregation ratio of 1:2:1 (low, intermediate and high F_dicyclic_) characteristic of a codominant monogenic model was accepted (χ^2^ = 2.65; threshold for accepting H_0_ at *P* = 0.05 is 5.99).

Analysis of F_C3_/F_C5_ values within locus *B* genotypes revealed *B*_CBDAS_*B*_CBDAS_-, *B*_THCAS_*B*_CBDAS_- and *B*_THCAS_*B*_THCAS_-specific distribution patterns (Fig. 4a–d, Supplementary Fig. S1). For *B*_CBDAS_*B*_CBDAS_ and *B*_THCAS_*B*_CBDAS_ genotypes, quadripartite distributions could be discerned from F_C3_/F_C5_ values (Fig. 4b,c). The most obvious deviation from the F_2_ F_C3_/F_C5_ distribution pattern was observed in the *B*_THCAS_*B*_THCAS_ genotypes, with KDE describing a tripartite distribution (Fig. 4d). Analogous with the complete F_2_ population (Fig. 3a), *B*_THCAS_*B*_CBDAS_ genotypes had a F_C3_/F_C5_ chemotype distribution resembling a composite of locus *B* homozygote inheritance patterns (Fig. 4b–d).

Given the high frequency of F_C3_ minima chemotypes among the F_2_ progeny (Figs 3a, 4b–d), complete dominance at one or more *A* gene pair locus was considered plausible for F_C3_/F_C5_ inheritance. Epistasis was also evaluated for locus *B* genotype-specific F_C3_/F_C5_ segregation ratios due to their non-conformity with Mendelian expectations. For the *B*_CBDAS_*B*_CBDAS_-specific F_C3_/F_C5_ quadripartite distribution pattern, a segregation ratio of 9:3:3:1 was accepted in support of a digenic model describing two independent Mendelian loci (Table 3). The *B*_THCAS_*B*_THCAS_-specific tripartite F_C3_/F_C5_ distribution conformed to a 7:6:3 segregation ratio, and an epistatic model describing dominance at one gene pair and partial dominance at the alternative gene pair was accepted (Table 3). A 9:3:3:1 segregation ratio was not supported by the *B*_THCAS_*B*_CBDAS_-specific F_C3_/F_C5_ values. Given the quadripartite nature of *B*_THCAS_*B*_CBDAS_-specific F_C3_/F_C5_ distributions, a 7:6:3 segregation ratio could not be tested (Table 3). *B*_THCAS_*B*_CBDAS_-specific F_C3_ categories I, II and IV did, however, share similar relative frequency and F_C3_/F_C5_ spacial distribution as the *B*_THCAS_*B*_THCAS_-specific categories (Table 3, Fig. 4c,d).

## Discussion

The contiguous pentapartite distributions in the F_2_ generation for F_C3_/F_C5_ values were not consistent with a polygenic binomial inheritance pattern. Quantitative characters are not exclusive to polygenic modes of inheritance^44,45^. Simple Mendelian inheritance can result in phenotypic continuity when within-genotypic class variation is large and average phenotypic differences between genotypes are negligible^45^. Given that alkyl-cannabinoid loci are associated with enzymatic reactions which are several biosynthetic steps upstream of the metabolites used for chemotypic assessment^46,47^, there is potential for intracellular biophysical interactions affecting the channelling and metabolic flux of pathway intermediates. Formation of multienzyme complexes has been implicated in altering isoprenoid production in *Arabidopsis thaliana* due to physical interactions between geranylgeranyl diphosphate (GGPP) synthase and downstream GGPP-consuming enzymes^48^. These interactions could affect the expression of alkyl cannabinoids and contribute to the continuous variation in chemotype values observed in filial populations.

The multi-model segregation pattern within the F_2_ progeny did not support a monogenic model, and so digenic inheritance was considered (Fig. 3a). In a digenic model with additive effects, a segregation ratio of 1:4:6:4:1 is expected^49^. However, F_C3_/F_C5_ values were skewed towards the F_C3_ minima parent and a disproportionate number of progeny segregated in the F_C3_ minima category (Fig. 3a, Table 3). Unequal additive effects at different loci associated with the alkyl cannabinoid pathway, combined with aggregation of trigenic heptapartite categories may also have contributed to an F_2_ chemotype segregation skewed towards the F_C3_ minima parent, although the frequency of F_C3_ maxima progeny in category V clearly exceeds the 1/64 allowed by this model (Table 3).

The inheritance of phenotypic traits can be additive or non-additive^50^. If the inheritance of genes indicates an additive effect, the hybrid phenotype will tend to reflect the average effect of the parent genes or midparent value (MPV)^50,51^. Phenotypic traits which deviate from the MPV in hybrid progeny are assumed to be inherited in a non-additive manor^45,50^, and inheritance can be attributed to dominant or epistatic gene effects^52^. Alkyl cannabinoid proportions within F_1_ family EIO.MW17.Y1, from which the F_2_ generation was derived, showed a negative median deviation from the MPV (44.6% F_C3_), with hybrid progeny displaying a median F_C3_ value of 35.1 (±6.7 s.d.) (range 24.3–56.0) % (Fig. 2a). Incomplete dominance and/or epistasis may therefore explain the deviation of EIO.MW17.Y1 chemotypes towards the F_C3_ minima parent (Fig. 2a). A non-additive model may also explain the higher frequency of F_C3_ minima progeny observed in the F_2_ generation (Fig. 3a).

Single seed descent F_8_ recombinant inbred lines as well as doubled haploid lines can achieve more than 99.7% homozygosity^53^. The parental lines used in the present study were not inbred to this level of homozygosity and parent heterozygosity may have contributed to the non-orthodox F_1_ and F_2_ inheritance patterns. Whilst the F_1_ family EIO.MW17.Y1 were descendants from parents displaying the largest F_C3_ divergence (Table 1), they also exhibited the highest level of F_C3_ homogeneity (Table 2), and displayed a uniform monopartite distribution largely consistent with a single category (Figs 2a, 3a). Taken together these factors suggest parental homozygosity at alkyl cannabinoid-determining loci. Given that within-plant C_3_/C_5_ alkyl cannabinoid composition has been found to be stable over key developmental stages, environmental and ontogenetic effects are also likely to have contributed minimally to inheritance patterns observed in the filial generations.

Secondary metabolite gene clusters comprising of two or more non-homologous biosynthetic pathway genes have been identified across a number of diverse plant taxa^54^. A common feature of these clusters is that they contain ‘signature genes’ in addition to other downstream pathway genes^54–56^. Signature genes are often recruited from primary metabolism and encode the first committed biosynthetic steps of the pathway^57^. For alkyl cannabinoid biosynthesis this is predicted to be the formation of alkylresorcinol fatty acid (FA) starter units^35,46,47^, which, when incorporated into the resorcinyl skeletal core^58^, influence directly carbon number of the resulting cannabinoid alkyl side-chain^37^. While the arrangement of cannabinoid synthesis pathway genes appear to be randomly dispersed over five chromosomes^39^, the enzymatic basis for cannabinoid FA starter unit synthesis, as well as genomic positioning of associated loci has yet to be established^39,46,47,59^. Given that cannabinoid synthase loci have been localised to retrotransposon-rich genomic regions compatible with gene cluster formation^39,40^, it is conceivable that upstream alkyl cannabinoid-determining loci may be physically clustered and/or co-inherited with *THCAS* and *CBDAS* genomic intervals.

The contrasting segregation ratios identified in *CBDAS* (*B*_CBDAS_*B*_CBDAS_) and *THCAS* (*B*_THCAS_*B*_THCAS_) homozygote F_2_ progeny suggests the possibility of linkage between alkyl and cyclic chemotype-determining loci and may explain the distortion of alkyl cannabinoid ratios from a strictly additive polygenic model (Fig. 4a–d, Table 3). Rearrangement of *THCAS* and *CBDAS* genomic regions is evident in the experimental population from the incomplete dominance and irregularity of the intermediate chemotypic distribution (Fig. 3b). Incomplete linkage between the SCAR markers and tandem cannabinoid synthase arrays may have precipitated synthetic genotype-specific inheritance patterns, although uncoupling of the marker with functionally relevant loci is questionable given that genotypes were largely congruent with chemotypic distributions (Fig. 4a). The association of the SCAR marker assay with chemotype has also been established across a range of geographically and genetically divergent *Cannabis* germplasm^60,61^.

*In vitro* feeding studies indicate that THCAS and CBDAS exhibit different catalytic efficiencies towards alkyl homologs^62^. This could be contributing to genotype-specific segregation patterns, although absence of appreciable levels of CBGA at UV 272 nm in filial F_C3_ maxima chemotypes would suggest otherwise (Supplementary Fig. S2). The UV profiles of F_C5_ plants were also dominated by CBDA and/or THCA and no comparable chromatographic peaks with a UV maxima and retention time consistent with CBGVA were observed. Whilst this would infer that cannabinoid synthases are capable of efficiently catalysing CBGA and CBGVA, it is conceivable that the affinity of alkyl homologs to THCAS and CBDAS is influencing the metabolic flux of oxidative cyclisation end-products, and hence the non-Mendelian inheritance patterns observed in filial chemotypes.

In the *CBDAS* homozygote (*B*_CBDAS_*B*_CBDAS_) F_2_ genotypes, the 9:3:3:1 ratio could be represented by *A*_C5_^1^ and *A*_C5_^2^ dominant and *A*_c3_^1^ and *A*_c3_^2^ recessive alleles, with double recessive genotypes *A*_c3_^1^*A*_c3_^1^*A*_c3_^2^*A*_c3_^2^ resulting in F_C3_ maxima chemotypes. Aliphatic glucosinolate side-chain length in *Brassica oleracea* is also regulated in a similar manner by independent assortment of *GSL-PRO* and *GSL-ELONG*^63^. The 7:6:3 ratio identified in *THCAS* homozygote F_2_ genotypes describes a more complex model, with dominance at one gene pair, and partial dominance at a second gene pair^64^. When homozygous recessive (*A*_c5_^1^ *A*_c5_^1^), the first gene pair is epistatic to the second gene pair^64^. Interestingly, a tripartite F_C3_/F_C5_ alkyl cannabinoid distribution was also identified from cluster analysis of a diversity panel comprised of predominantly tricyclic cannabinoid chemotypes^15^.

**Figure 5:**
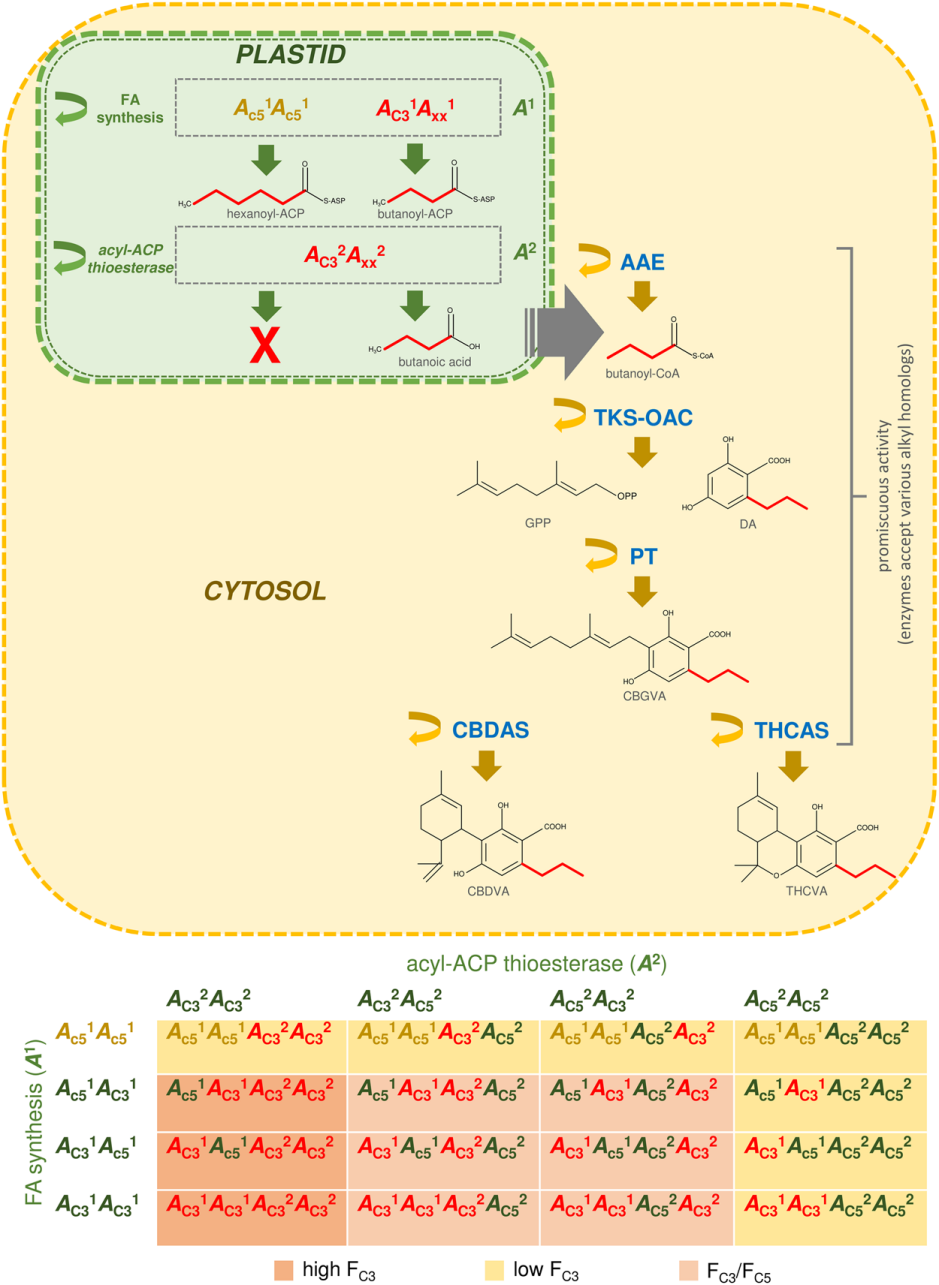
Speculative digenic epistatic model governing alkyl cannabinoid composition in *THCAS* homozygote plants. The dominant *A*_C3_^1^ allele at the first gene pair governs one of four condensing, reductase or dehydrase reactions forming butanoyl-ACP. The partially dominant allele *A*_C3_^2^ at the second gene pair encodes an acyl-ACP thioesterase with high catalytic efficiency (kcat) for butanoyl-ACP. The acyl-ACP thioesterase allows plastid exportation of butanoic acid for cytosolic-localised alky cannabinoid biosynthesis. The homozygous recessive genotype (*A*_c5_^1^ *A*_c5_^1^) at the first gene pair results in the exclusive production of hexanoic acid and is epistatic to the second gene pair encoding the 4:0-ACP thioesterase; acyl activating enzyme (AAE); acyl-acyl carrier protein (ACP); cannabidiolic acid synthase (CBDAS); cannabidivarinic acid (CBDVA); cannabigerovarinic acid (CBGVA); divarinolic acid (DA); fatty acid (FA); geranyl pyrophosphate (GPP); olivetolic acid cyclase (OAC); prenyltransferase (PT); delta(9)-tetrahydrocannabinolic acid synthase (THCAS); delta(9)-tetrahydrocannabivarinic acid (THCVA); tetraketide synthase (TKS); C_5_ alkyl cannabinoid fraction (F_C5_); C_3_ alkyl cannabinoid fraction (F_C3_).

One speculative scenario to describe the aforementioned epistatic model is that *THCAS* co-inherited alkyl cannabinoid loci encode sequential interdependent enzymatic steps^65,66^ (Fig. 5). *De novo* short-chain FA synthesis *in planta* is dependent on a series of enzymatic reactions involving β-ketoacyl-ACP synthase, β-ketoacyl-ACP reductase, β-hydroxyacyl-ACP dehydrase as well as enoyl-ACP reductase^67^, followed by thioesterase hydrolysis to terminate synthesis^68^. The dominant *A*_C3_^1^ allele at the first gene pair may govern one of four condensing, reductase or dehydrase reactions which contribute towards FA chain length^69^, resulting in increased production of butanoyl-ACP (Fig. 5). *A*_C3_^2^ at the second gene pair could encode a thioesterase with high catalytic efficiency (kcat) towards butanoyl-ACP, thereby allowing FA plastid exportation of butanoic acid for downstream cytosolic-localised alkylresorcinol synthesis^47,68^ (Fig. 5). The *A*_C3_^2^ modifier would act only on butanoyl-ACP and when homozygous recessive for *A*_c5_^1^, FA synthesis would be exclusive to the C_5_ alkyl cannabinoid precursor hexanoic acid (Fig. 5).

Previous analysis of six S_1_ to S_6_ inbred lines segregating for tricyclic C_3_ and C_5_ alkyl cannabinoids revealed a variety of lineage-specific distribution patterns^31^. A polygenic inheritance model was inferred from the absence of 100% ‘pure’ C_3_-alkly cannabinoid chemotypes as well as from the mutual crossing of lineages increasing C_3_ alkyl cannabinoid proportion from 85.5–95.6%^31^. In the present study, digenic inheritance patterns were adequate to explain F_C3_ values ranging from 0.7–88.0% (Table 1, Fig. 4a).

Absence of F_C3_ transgressive segregation or plants displaying F_C3_ values > 90% suggests a chemotypic plateau has been reached in the experimental population (Table 1), and that parental genes lack a complementary additive effect on F_C3_ values^70^. A number of enzymatic reactions occur prior to oxidative cyclisation by THCAS and CBDAS. These involve a series of steps leading to FA formation in addition to acyl activation^47^, two-step polyketide synthesis^38^ and aromatic prenylation^33^ (Fig. 5), of which a minimum of two catalytic steps were found to be allelic and determinant of chemotype (Table 3, Fig. 4b,d). Analysis of cannabinoid biosynthesis in engineered yeast indicates that acyl activation, polyketide synthesis and aromatic prenylation steps are catalysed by promiscuous enzymes, with recombinant pathway proteins capable of producing a variety of alkyl homologs based on the type of FA starter unit fed^37^. Assuming cannabinoid pathway loci are allelic and encode enzymes with varying levels of promiscuity^37^, gene-flow at these loci may confer an additive or epistatic effect and a polygenic alkyl cannabinoid inheritance model may be correct. However, with consideration of measurement error and environmental deviation^15,71^, the lineage-specific gene effects reported from mutual crossing may only be marginal.

Regardless of the total number of loci contributing to alkyl-cannabinoid composition, inheritance patterns reported here and elsewhere suggest the partitioning of allelic variation among lineages^31^. Inter-lineage genetic heterogeneity has the potential to confound elucidation of the genetic architecture underlying alkyl cannabinoid composition when using forward genetic approaches. Quantitative Trait Locus (QTL) mapping may only capture a subset of inter-lineage allelic diversity and associated epistasis in natural populations^72^, while in Genome Wide Association Studies (GWAS), genetic heterogeneity among lineages reduces the power to detect causal variants^73,74^. In these cases, synthetic and/or ancestral marker loci may be more predictive of phenotype when two or more gene mutations have a comparable phenotypic effect^74^. Given that lineage-specific evolutionary processes are implicated at cannabinoid pathway loci^40^, comparative genomic approaches using representative germplasm may precipitate diagnostically valuable chemotype-associated markers while also potentially delineating candidate alkyl cannabinoid loci for genome engineering^8^.

The analysis of filial chemotypes was targeted towards variation in alkyl side-chain length. Whilst this analysis improved understanding of the heritability of cannabinoid homology, much remains to be examined. In addition to variation in the topological arrangement of the isoprenoid residue^13^, prenylogous versions of cannabinoids have been identified in the form of sesquicannabigerol^75^. This degree of isoprenylation improved pharmacological potency towards CB_2_R and it is possible that other medically relevant cannabinoid prenylogues may exist^75^. Further non-targeted cannabinomic analyses, combined with forward genetic screens, may further elucidate the molecular basis for cannabinoid homology and ultimately expand the number of therapeutics which can be produced *in planta*.

In conclusion, the inheritance of alkyl cannabinoid composition and associated allelic assortment with *THCAS* and *CBDAS* was examined. Digenic segregation patterns observed in cannabinoid synthase genotypes suggests a complex mode of inheritance for alkyl side-chain length involving epistasis, linkage as well as dominant and lineage-specific gene effects. Linking plant secondary metabolites to underlying biosynthetic genes and associated regulatory networks remains challenging and often requires a multifaceted approach^76,77^. Comparative genomic approaches may contribute to understanding of the molecular basis for alkyl cannabinoid composition and shed light on the recruitment and evolution of pathway genes. Advances in understanding of the inheritance and biosynthesis of the alkyl pharmacophore may also allow for metabolic engineering of *Cannabis* to accelerate development of novel efficacious plant-derived cannabinoid homologs with augmented therapeutic activities.

## Methods

### Genetic resources and cultivation

Acquisition and storage of research materials and associated experimental procedures were conducted under the provisions of the Drug Misuse and Trafficking Act 1985 and in accordance with authorisations granted to Professor Graham King by the New South Wales Ministry of Health, Pharmaceutical Regulatory Unit, Legal and Regulatory Services Branch, Australia. Three *Cannabis sativa* L. seed pack accessions EIO.MW15.P, EIO.MW15.T and EIO.MW17.X associated with either high C_3_ alkyl (F_C3_) tricyclic (F_tricyclic_) or high C_5_ alkyl (F_C5_) dicyclic (F_dicyclic_) compositions were sourced from the Ecofibre Industries Operations Pty Ltd Global Germplasm Collection (EFGGC) (Table 1).

Twenty seeds per accession were sown into 400 mL round pots at a depth of 1.5 cm. Each pot contained a growing medium containing one-part vermiculite, one-part perlite and one-part peat moss, as well as dolomite (110 g/100 L). Pots were watered daily and supplemented with CANNA^®^ Aqua Vega nutrient solution post germination upon full extension of the first leaflet pair. Seedlings were grown indoors within bespoke pollen secure growth chambers and grown under an 11 h photoperiod using high pressure sodium (HPS) and metal halide (MH) lighting (luminous flux = 72,000 lumens). At the flower primordia stage (code 2001)^78^, selected plants were transferred into single 8 L pots containing 100 g Osmocote^®^ Exact slow release nutrient mix and 8 g of Micromax^®^ micronutrient formula. Optimal water regimes were controlled using automatic ‘smart valves’ and temperature was maintained between 26 and 28 °C.

### Experimental populations

Individual plants from accessions EIO.MW15.P (*n* = 22), EIO.MW15.T (*n* = 18), EIO.MW17.X (*n* = 13) were screened for chemotype using LC-MS cannabinoid profiling at the vegetative stage (code 1008)^78^ (Table 1). Plants which exhibited high F_C3_ and F_tricyclic_ (e.g. THCVA) or high F_C5_ and F_dicyclic_ (e.g. CBDA) cannabinoid values were selected for crossing (Table 1, Fig. 1). Sex was provisionally phenotyped from visual inspection during the flower primordia developmental stage prior to male anthesis (code 2001)^78^. Plant vigour was also considered during selection. Eight chemotypically extreme male and female plants high in THCVA (EIO.MW15.P) or CBDA (EIO.MW15.T and EIO.MW17.X) served as parents for four F_1_ hybrid families, which were generated from two biparental reciprocal crosses (Table 1, Fig. 1). Generation of 210 F_2_ progeny was achieved by crossing a single male and female plant from the F_1_ hybrid family which exhibited the highest level of F_C3_ chemotypic homogeneity. Biparental crosses were performed within pollen secure growth chambers. Pollination of female plants was achieved through exposure to male plants during anthesis.

### LC-MS chemotyping

Liquid chromatography-mass spectrometry (LC-MS) cannabinoid profiling and extraction of individual plants followed methodologies described by Welling *et al*.^15^. At the vegetative stage (fourth leaf pair, code 1008)^78^, two × 250 mg fresh leaf material was taken from the sub-apical raceme at opposing phyllotaxis. Plant material was transferred to a 2 mL Eppendorf^®^ Safe-Lock microcentrifuge tubes containing a 3 mm Qiagen Tungsten Carbide Bead and frozen using liquid nitrogen. Plant tissue was disrupted using a Qiagen TissueLyser^®^ by agitation at 30 rotations per sec for 60 s. Plant tissue was vortexed in 1 mL of high-performance liquid chromatography (HPLC) grade EtOH and mixed by agitation for 30 min. Extracts were centrifuged to remove particulate matter and 600 μL of the supernatant was transferred into a 2 mL screw cap glass vial.

LC-MS runs were performed using an Agilent 1290 Infinity analytical HPLC instrument (Agilent Technologies, Palo Alto, CA, United States), which comprised of a vacuum degasser, autoinjector, binary pump and diode array detector (DAD, 1260), coupled to an Agilent 6120 Single Quadrupole Mass Selective Detector (MSD). Analytical infrastructure was controlled using ChemStation (Agilent) software (Rev. B.04.03 [54]). A C_18_ Agilent Eclipse plus rapid resolution high definition column (1.8 μm; 50 mm × 2.1 mm internal diameter) was used. Absorbance was monitored at 210, 214, 272, 280, 330 and 360 nm.

The mobile phase consisted of a mixture of Milli-Q^®^ water (channel A) and acetonitrile (channel B) containing 0.005% trifluoroacetic acid (TFA). The initial setting was isocratic at 66% B for 8 min, which was linearly increased to 95% B over 4 min. 95% B was maintained for 1 min and then re-equilibrated to 66% B for 2 min. Total run time including an internal needle wash was 16 min. Flow rate was 0.3 mL/min. Column temperature was set to 30 °C. Injection volume was 3 μL. The MSD was run in atmospheric pressure electrospray ionisation mode (AP-ESI). Selected-ion monitoring (SIM) was used for cannabinoid quantification, with abundant and representative signals obtained in positive mode [M + H]^+^ ^15^; drying gas temperature, 350 °C; capillary voltage, 3000 V (positive); vaporiser temperature, 350 °C; drying gas flow, 12 L/min (N2); nebuliser pressure, 35 psi; scan mass range, 100–1200; fragmentor, 150.

Cannabinoid standards cannabinol (CBN), CBGA, cannabigerol (CBG), cannabidiolic acid (CBDA), CBD, cannabidivarin (CBDV), cannabichromene (CBC), delta(9)-tetrahydrocannabinolic acid (THCA), THC, and delta(9)-tetrahydrocannabivarin (THCV) were sourced from Novachem Pty Ltd. (Melbourne, VIC, Australia). THCVA and CBDVA were developed in-house using an Agilent 1260 Infinity preparative HPLC system, with purified fractions structurally elucidated using a Bruker Avance III HDX 800 MHz spectrometer^15^. Calibration solutions for acidic as well as neutral reference cannabinoids were prepared at 100, 20, 4, 0.8, 0.16, 0.032 μg/mL and calibration curves for each cannabinoid were linear across the calibration range (r^2^ > 0.99). Precision was determined by injecting stock solutions six times and monitoring cannabinoid peak area (relative standard deviation (RSD) < 2%). Interday MSD variability was minimised by running calibration curves every 48 hours. Data acquisition and analysis was performed using Agilent ChemStation^©^ (Rev. B.04.03 [54]) software.

### Locus *B* DNA SCAR marker

Plant DNA was extracted using a Qiagen DNeasy^®^ Plant Mini Kit, with tissue disruption achieved using a Qiagen TissueLyser^®^. DNA purity was assessed using a ThermoScientific^TM^ NanoDrop^TM^ 2000 UV–vis spectrophotometer. An absorbance ratio of ~1.8 at 260/280 nm and symmetric peaks at 260 nm were used to determine DNA quality.

Amplification of *CBDAS* (B1080) and *THCAS* (B1190) sequence characterised amplified region (SCAR) fragments was accomplished using a *B* locus-specific multiplex PCR assay comprising of three primers: a primer common to *CBDAS* and *THCAS* FW: 5′ AAGAAAGTTGGCTTGCAG 3′ as well as a *CBDAS*-specific REV: 5′ ATCCAGTTTAGATGCTTTTCGT 3′ and a *THCAS*-specific REV: 5′ TTAGGACTCGCATGATTAGTTTTTC 3′ primer^60,79^.

PCR parameters followed those described by Welling *et al*.^61^. Reactions were performed in 0.2 mL 96 well PCR plates in a total volume of 50 µL and contained 1.5 mM of MgCl_2_, 0.2 mM of dNTPs, 0.4 µM of the forward primer, 0.2 µM of the *THCAS*- as well as the *CBDAS*-specific reverse primers, and 2 U of Life Technologies Platinum^®^ Taq DNA Polymerase. Thermocycling parameters for the DNA template were as follows: 94 °C for 2 min, followed by 25 cycles of 94 °C for 30 s, 58 °C for 30 s, 72 °C for 1 min 15 s. No final extension was required. *CBDAS*- and *THCAS*-specific fragments were then separated using electrophoresis with a 1% SeaKem^®^ LE agarose gel stained with GelRed^TM^. Amplicons were visualised under UV illumination with a Bio-Rad Molecular Imager^®^ Gel Doc^TM^ XR + system using Image Lab^TM^ software.

### Statistical analysis

CBDVA, THCVA, CBDA, THCA, CBDV, THCV, CBD and THC fresh weight (*w/w*) content was determined per plant. Relative proportions of these cannabinoids was used to generate C_3_ alkyl (F_C3_), C_5_ alkyl (F_C5_), dicyclic (F_dicyclic_) and tricyclic (F_tricyclic_) cannabinoid fractions within the total cannabinoid fraction. To minimise post-harvest alteration of cannabinoid composition, decarboxylated cannabinoids CBDV, THCV, CBD and THC were expressed as carboxylated acid (COOH) cannabinoids using formulae which compensate for changes in molecular weight^15^. Repeatability between LC-MS replicate extractions were calculated using coefficient of determination (r^2^). Strong correlations between duplicate extraction replicates were found for the F_C3_/F_C5_ (r^2^ > 0.99) as well as for the F_dicyclic_/F_tricyclic_ (r^2^ > 0.99) values. Mean extraction replicate values were therefore used for statistical analysis.

Alkyl cannabinoid data from the F_2_ generation was visualised in a graphical format used previously^31^ (Supplementary Fig. S3). Analysis of F_2_ chemotypic distribution patterns revealed stepwise increases in F_C3_ values, although accurate demarcation of data points was not possible (Supplementary Fig. S3). Histograms were then developed to establish frequency distributions for categorisation (Supplementary Fig. S3). However, the continuity of chemotype prevent formation of obvious break points in the data (Supplementary Fig. S3). The arbitrary selection of bins was also deemed inappropriate for determining distributions due to the potential for incorrect assignment of genotype (classification error). To address these issues, kernel density was used to estimate the unknown underlying distributions within the data. This constructed an estimate of the density function from observations within the data^43^, generating a fitted solid line over the F_C3_ value data points (Supplementary Fig. S3). The area under kernel density estimates (KDE) was then used to demarcate F_C3_ values and to objectively categorise plants (Supplementary Fig. S3), circumventing arbitrary categorisation and the artificial grouping of F_C3_ values.

GenStat 64-bit Release 18.1 (VSN International Ltd.) software was used to calculate Bartlett’s test for homogeneity of variances, KDE and Pearson’s chi-squared (χ^2^) goodness-of-fit. For KDE, automatic estimation of the bandwidth *h* was achieved using the method proposed by Sheather and Jones^80^. Kernels supported by a frequency of *n* = 1 were not considered. Categorisation frequencies for Pearson’s χ^2^ goodness-of-fit were obtained by baseline peak integration of KDE, which provided chemotypic grid point inclusion/exclusion boundaries (Supplementary Fig. S3).

## Supplementary information

Supplementary Information

## Data Availability

The LC-MS datasets derived from experimental populations generated and/or analysed during the current study are available from the corresponding author on reasonable request.
