# Supplementary material for: Complex Patterns of Cannabinoid Alkyl Side-Chain Inheritance in Cannabis
Source: Sci Rep. 2019 Aug 6;9:11421. doi: 10.1038/s41598-019-47812-2 (PMC6684623; doi:10.1038/s41598-019-47812-2)
Supplement: Supplementary file 1 — Supplementary Information [file 41598_2019_47812_MOESM1_ESM.pdf]

Supplementary Information

Complex Patterns of Cannabinoid Alkyl Side-Chain Inheritance in *Cannabis*

Matthew T. Welling[1,2] , **Lei Liu**[1] , **Carolyn A. Raymond**[1] , **Tobias Kretzschmar**[1] , **Omid Ansari**[2,3] , **Graham J. King**[1,*]

[1]Southern Cross Plant Science, Southern Cross University, Lismore, New South Wales 2480, Australia.

[2]Ecofibre Ltd, Brisbane, Queensland 4014, Australia.

[3]Ananda Hemp Ltd, Cynthiana, Kentucky 41031, USA.

*graham.king@scu.edu.au

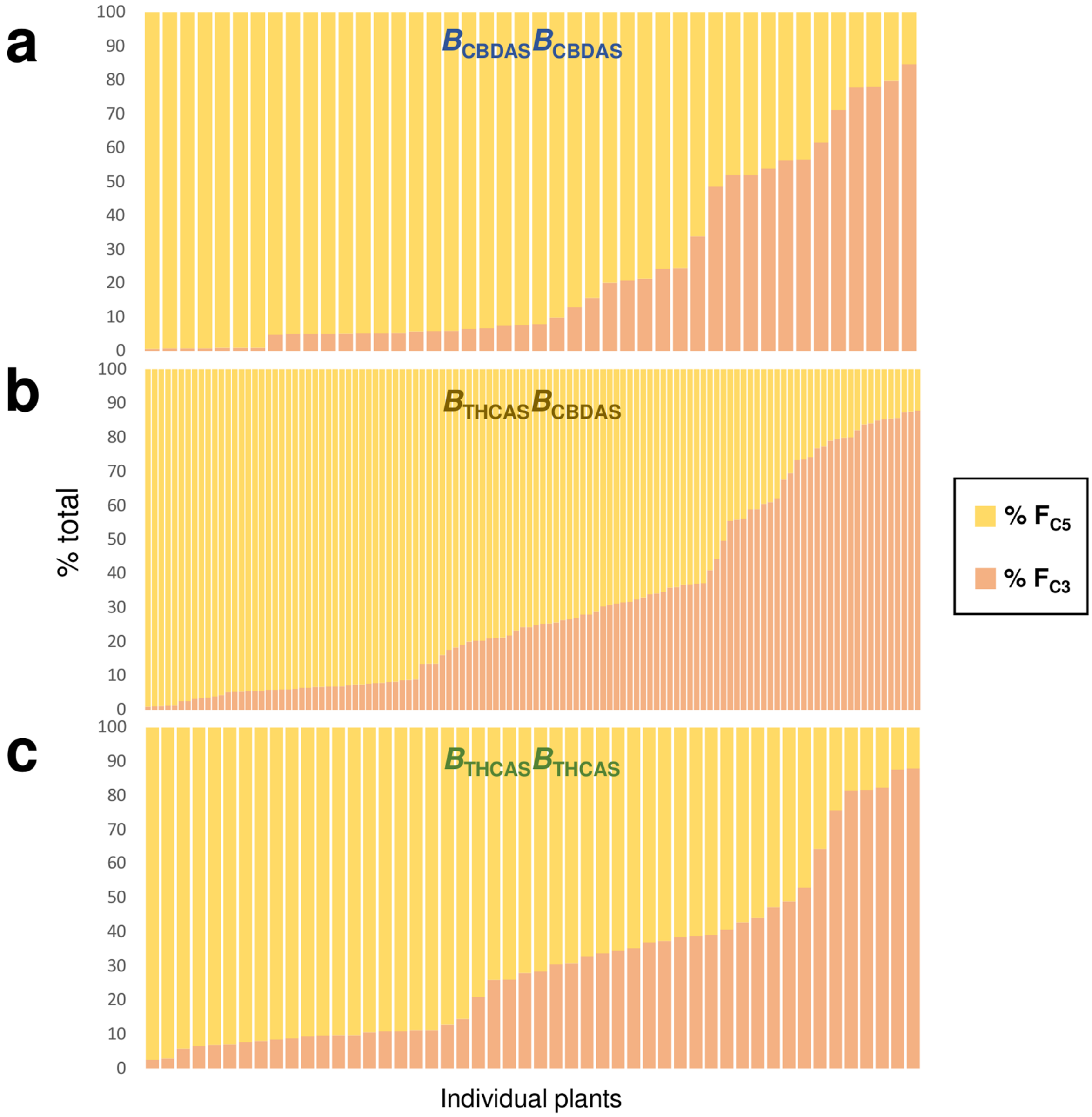

Supplementary Figure S1. Alkyl cannabinoid inheritance patterns of locus *B* genotypes. (a) Bar graph of $B_{CBDAS}B_{CBDAS}$ alkyl cannabinoid chemotypes. (b) Bar graph of $B_{THCAS}B_{CBDAS}$ alkyl cannabinoid chemotypes. (c) Bar graph of $B_{THCAS}B_{THCAS}$ alkyl cannabinoid chemotypes. Individual plant chemotypes on the *x*-axis ordered from low to high F_{C3}. C_5-alkyl cannabinoid fraction (F_{C5}); C_3-alkyl cannabinoid fraction (F_{C3}); locus *B* genotypes: homozygote *THCAS* ($B_{THCAS}B_{THCAS}$), homozygote *CBDAS* ($B_{CBDAS}B_{CBDAS}$), heterozygote *THCAS CBDAS* ($B_{THCAS}B_{CBDAS}$)

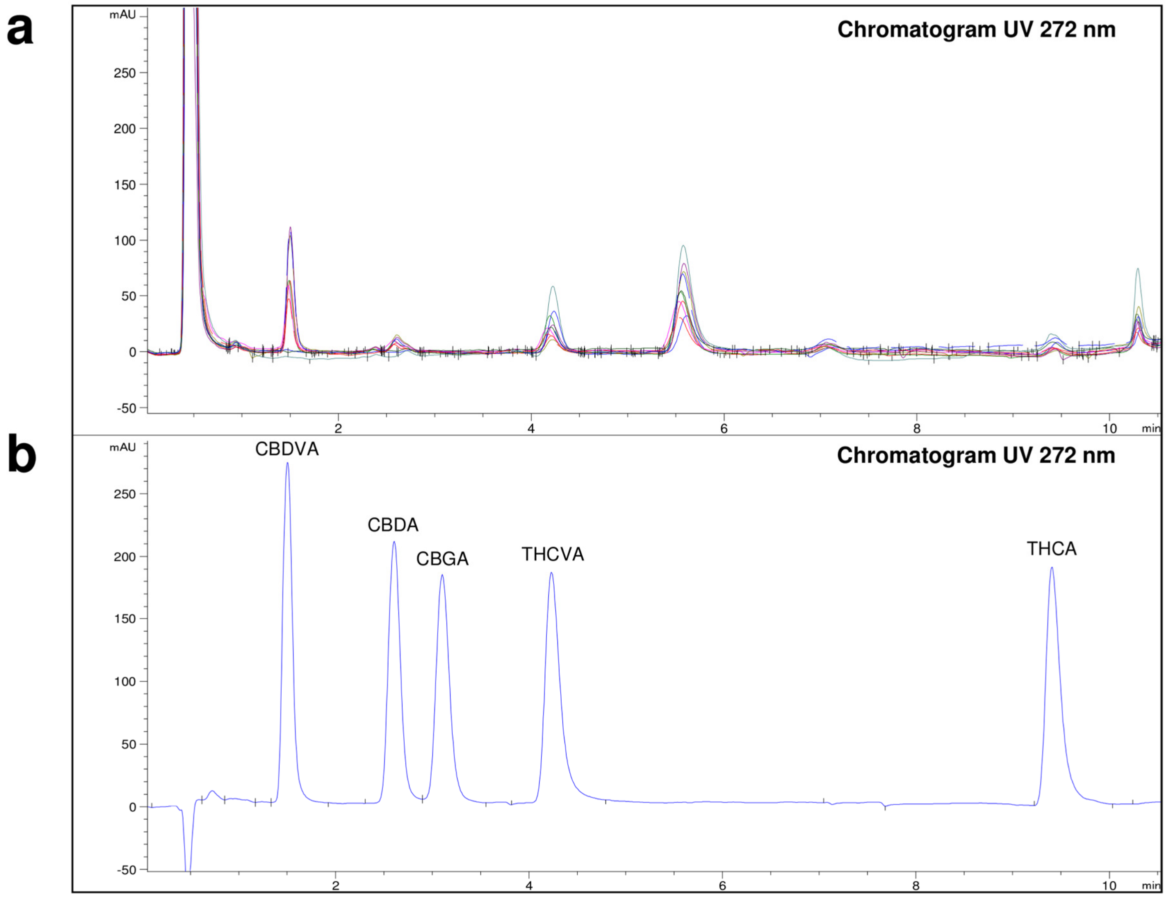

Supplementary Figure S2. Chromatograms showing C_3 and C_5 alkyl cannabinoids. (a) Overlay of exemplar chromatograms from F_2 progeny exhibiting C_3-alkyl cannabinoid maxima chemotypes. Appreciable levels of CBGA not present in the profile of CBDVA/THCVA predominant chemotypes. (b) Chromatogram of alkyl cannabinoid analytical standards. Cannabidiolic acid (CBDA); cannabidivarinic acid (CBDVA); cannabigerolic acid (CBGA); delta(9)-tetrahydrocannabinolic acid (THCA); delta(9)-tetrahydrocannabivarinic acid (THCVA)

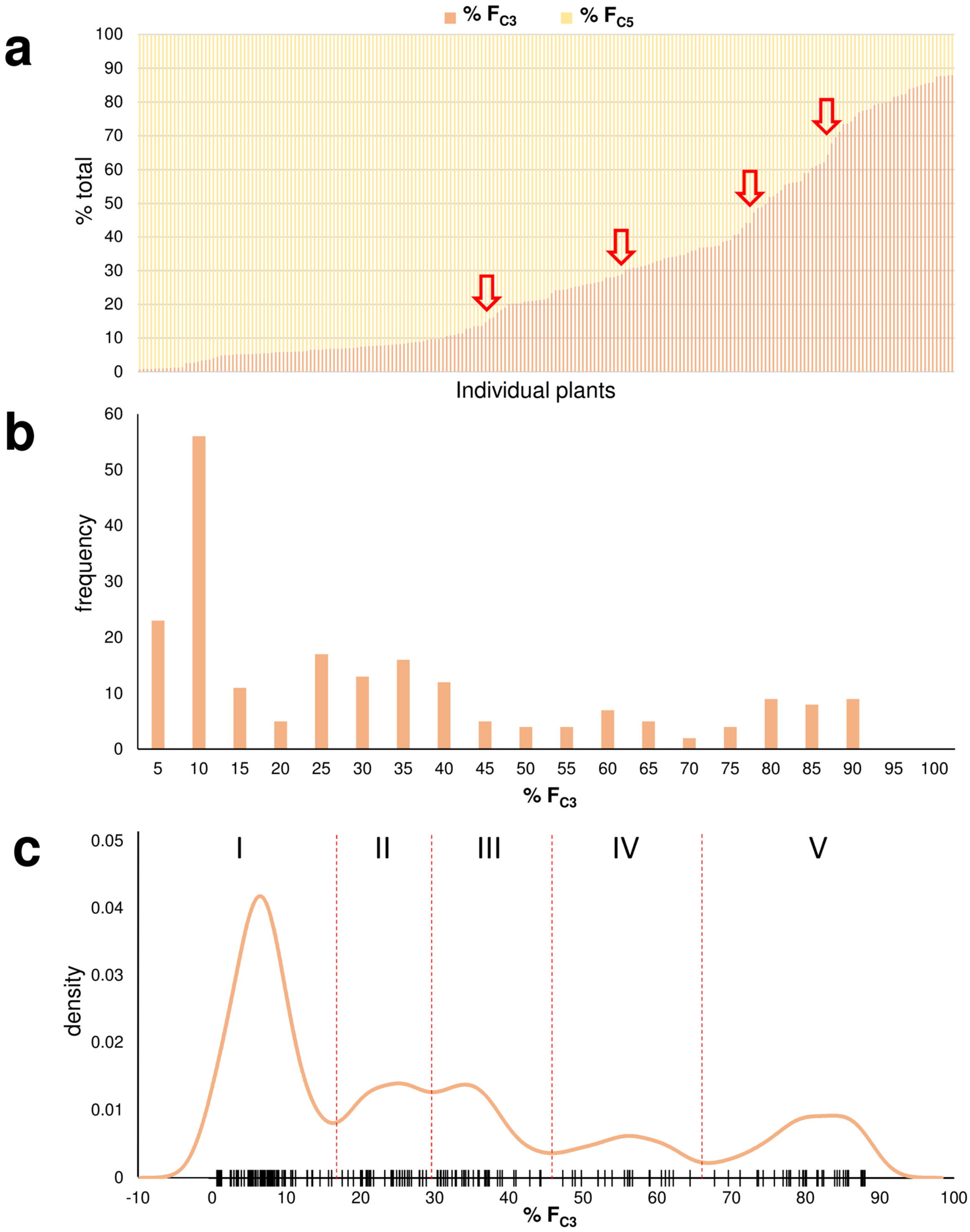

Supplementary Figure S3. Inheritance patterns of alkyl cannabinoid composition. (a) Bar graph of alkyl cannabinoid chemotypes of individual plants showing stepwise increases in F_{C3} value. Individual plant chemotypes on the *x*-axis ordered from low to high F_{C3}. (b) Histogram of alkyl cannabinoid chemotypes showing discrete F_{C3} frequency distributions. (c) Kernel density estimation of F_{C3} values objectively demarcating data points in to five chemotypic categories. *Black lines* indicate individual plant F_{C3} values. C_5-alkyl cannabinoid fraction (F_{C5}); C_3-alkyl cannabinoid fraction (F_{C3})
